# The Optimal Second-Line Systemic Treatment Model for Recurrent and/or Metastatic Head and Neck Squamous Cell Carcinoma: A Bayesian Network Meta-Analysis

**DOI:** 10.3389/fimmu.2021.719650

**Published:** 2021-08-02

**Authors:** Ze-Jiang Zhan, Wen-Yu Yao, Fang Zhang, Wen-Ze Qiu, Kai- Liao, Jian-Hui Feng, Jin-Yun Tan, Hui Liu, Tai-Ze Yuan, Rong-Hui Zheng, Ya-Wei Yuan

**Affiliations:** ^1^Department of Radiation Oncology, Affiliated Cancer Hospital and Institute of Guangzhou Medical University, Guangzhou, China; ^2^Health Ward, Affiliated Cancer Hospital and Institute of Guangzhou Medical University, Guangzhou, China; ^3^Department of Radiation Oncology, Guangzhou Concord Cancer Center, Guangzhou, China

**Keywords:** recurrent and/or metastatic head and neck squamous cell carcinoma, treatment, efficacy, toxicity, network meta-analysis

## Abstract

**Background:**

The optimal second-line systemic treatment model for recurrent and/or metastatic head and neck squamous cell carcinoma (R/M HNSCC) remains controversial. A Bayesian network meta-analysis (NMA) was performed to address this issue with regard to efficacy and toxicity.

**Methods:**

By searching MEDLINE (*via* PubMed), Embase, the Cochrane Central Register of Controlled Trials and Web of Science, we extracted eligible studies. Efficacy, represented as overall survival (OS) and progression-free survival (PFS), and overall toxicity, represented as ≥ grade 3 severe acute events (sAE), were assessed to compare the following 7 treatment models through an NMA: standard-of-care therapy (SoC), single targeted therapy different from SoC (ST), double targeted therapy (DT), targeted therapy combined with chemotherapy (T+C), single immune checkpoint inhibitor therapy (SI), double immune checkpoint inhibitor therapy (DI) and single chemotherapy different from SoC (SC). Rank probabilities according to the values of the surface under the cumulative ranking curve (SUCRA) were separately determined for efficacy and toxicity.

**Results:**

In total, 5285 patients from 24 eligible studies were ultimately screened, with 5184, 4532 and 4026 involved in the NMA of OS, PFS and sAE, respectively. All qualifying studies were absent from first-line immune checkpoint inhibitor therapy. In terms of OS, SI was superior to the other treatments, followed by DI, ST, T+C, SoC, DT and SC. Other than SI and SC, all treatments tended to be consistent, with hazard ratios (HRs) close to 1 between groups. For PFS, ST ranked first, while DT ranked last. For the toxicity profiles, compared with the other models, SI resulted in the lowest incidences of sAE, with statistical significance over SoC (odds ratio [OR] 0.31, 95% credible interval [CrI] 0.11 to 0.90), ST (OR 0.23, 95% CrI 0.06 to 0.86) and DT (OR 0.11, 95% CrI 0.02 to 0.53), while DT was the worst. When the SUCRA values of OS and sAE were combined, a cluster plot illustrated the superiority of SI, which demonstrated the best OS and tolerability toward sAE.

**Conclusion:**

For R/M HNSCC patients without immune checkpoint inhibitors in the first-line setting, SI may serve as the optimal second-line systemic treatment model, demonstrating the best OS and least sAE.

## Highlights

This network meta-analysis, encompassing 5285 individuals from 24 trials which were absent from first-line immune checkpoint inhibitor therapy, revealed that single immune checkpoint inhibitor therapy achieved the best overall survival as well as the least severe acute events in the second-line treatment of R/M HNSCC patients who were unable or did not have access to immunosuppressants in the first-line setting. It provides recommendations for future clinical decision-making and trial designs.

## Introduction

Head and neck squamous cell carcinoma (HNSCC), originating from the epithelial tissue of the head and neck region, includes mainly oral cancer, oropharyngeal cancer, hypopharyngeal cancer and laryngeal cancer. As estimated by the International Agency for Research of Cancer, there were approximately 830,000 patients newly diagnosed with HNSCC worldwide in 2018, and approximately 430,000 patients died of this type of tumor throughout the year (1).

HNSCC is a biologically and clinically heterogeneous disease, and its prognosis is quite less than satisfactory (2). Approximately 4% ~ 26% of patients present with metastatic disease at the first diagnosis, and among patients who have not been diagnosed with metastasis, despite radical treatment, more than 30% will eventually develop recurrent and/or metastatic (R/M) diseases (3–8). Treatment options for R/M HNSCC are limited, especially in regard to second-line regimens, mainly referring to chemotherapy, targeted therapy or a combination of the two. Adoption of these regimens leads to median progression-free survival (PFS) and overall survival (OS) times of only 2 ~ 3 and 6 ~ 8 months, respectively (9–11). Encouragingly, the advent of immunotherapy has led to survival hope for patients with metastatic tumors in recent years (12). However, its application is controversial in the administration of different immune checkpoint inhibitors (ICIs) in HNSCC (13–16). In the CheckMate 141 and KEYNOTE 040 trials, the results showed that ICIs significantly increased OS over standard therapy as second-line treatments (13, 14). Therefore, both nivolumab and pembrolizumab have been recommended by the National Comprehensive Cancer Network (NCCN) guidelines. Notably, in the EAGLE trial, which explored the use of single and double ICIs as second-line regimens for R/M HNSCC, no statistically superior efficacy was observed with respect to standard therapy (16). This opposing outcome poses a challenge to the earlier research. In addition, dual ICIs exhibited no survival benefit over single applications in the EAGLE trial, which is consistent with the results of the CONDOR trial (15). Hence, controversy remains in regard to whether ICIs can be used as the optimal treatment and to how it should be used in combinations.

KEYNOTE 048 is a notable study evaluating the efficacy of pembrolizumab monotherapy or with chemotherapy in comparison to the Extreme regimen, which marked a remarkable survival superiority when pembrolizumab was administered (17). Its advent facilitates the widespread application of pembrolizumab in the first-line treatment of R/M HNSCC, however, pembrolizumab is presently not so affordable and there are many developing countries where ICIs are not yet approved in the front line setting and only in the second-line setting owing to the fact that treatment with pembrolizumab-related regimens is a cost-effective strategy in the developed country like the USA, whereas in the developing country like China, it is the opposite and the Extreme regimen still stands out as a cost-effective choice (18, 19). In patients who have experienced ICIs in the first-line setting, the subsequence choices will definitely catch public attention, however, this would be a longer-than-expected event in certain patient populations since preferring pembrolizumab in the first-line is not a cost-effective strategy in certain developing countries (18, 19). Under these circumstances, in patients who are absent from first-line immunotherapy, the second-line choice concerning ICIs is still worthy of explorations. Further determination of the exact role immunotherapy plays in second-line treatment is imperative.

Since few trials have directly compared immunotherapy with other treatments, this further exploration of the best second-line systemic treatment model for R/M HNSCC can be addressed by conducting a network meta-analysis (NMA), which can directly and/or indirectly compare different groups from the current literature and, to a certain extent, make up for the deficiency in the limited treatment comparisons in clinical trials to date in order to determine the best treatment group (20). Therefore, by performing an NMA, we conducted this work to determine the optimal second-line systemic treatment model for R/M HNSCC in terms of efficacy and toxicity.

## Materials and Methods

Abiding by the Preferred Items for Systematic Reviews and Meta-Analyses (PRISMA) reporting guidelines as well as the extension statement of NMA (21, 22), this work was undertaken, and the relevant protocol was registered in INPLASY (No. 2020110041). The Institutional Review Board of Affiliated Cancer Hospital and Institute of Guangzhou Medical University granted this review exemption due to the innocuousness of this research, and no ethical approval was required. All necessary data were extracted from online reported studies on MEDLINE (*via* PubMed), Embase, the Cochrane Central Register of Controlled Trials and Web of Science. Two reviewers (Zhan ZJ, Zhang F) independently executed the subsequent search process. Whenever disagreements arose, a consultation referring to a senior investigator (Qiu WZ) was carried out to achieve a consensus.

### Literature Search

**Supplementary Table 1** describes in detail the search strategy for this NMA. First, up to October 2020, which was finally updated on June 2021, screening was performed *via* the relevant research titles and abstracts, and duplicated publications were removed. Then, for the assessment for final inclusion, we thoroughly browsed the full text of the articles as well as the corresponding reference lists. Those publications that did not strictly meet the following inclusion criteria were excluded.

### Study Selection

The inclusion criteria were as follows:

- Randomized controlled trials (RCTs);
- Patients with pathologically confirmed R/M HNSCC;
- Use of treatments including chemotherapy, targeted therapy, immunotherapy or a combination thereof;
- Survival data and/or toxicity profiles available in the study or could be calculated with the relevant reported data.

Exclusion criteria were as follows:

- Pathologically confirmed nasopharyngeal carcinoma as the only type of tumor included in the whole cohort;
- Use of local treatments such as surgery and radiotherapy in a second-line setting;
- A second primary HNSCC;
- Studies in which toxicity profiles were not presented as ≥ grade 3 toxicity overall based on National Cancer Institute-Common Terminology Criteria for Adverse Events (NCI-AECTC) (available at http://ctep.cancer.gov);
- Studies in which full-text reports were unavailable.

### Data Extraction and Risk of Bias Assessment

We collated the relevant data from all eligible studies onto an electronic spreadsheet, which mainly included first author, year of publication, sample sizes, institutions, trial design, interventions and outcome measures. Hazard ratios (HRs) were determined for efficacy outcome assessments of OS/PFS, and odds ratios (ORs) were used for safety profile measures of treatment-related severe acute events (sAE), along with their 95% credible intervals (CrI). OS and PFS were defined as the interval from randomization to last follow-up or death and the interval from randomization to tumor progression or death, respectively. The sAE was defined as ≥ grade 3 overall toxicity. For studies lacking HRs, the Engauge Digitizer (version 4.1) and a calculation spreadsheet were utilized to estimate HRs through Kaplan-Meier curves along with their 95% CrI according to the methods described by Tierney and colleagues (23). If the related outcomes were unavailable in the study, an attempt was made to consult the study authors to obtain individual patient data of interest.

Adhering to the Cochrane Risk of Bias Tool (24), the risk of bias of the included RCTs was assessed. It includes 7 domains, namely, random sequence generation, allocation concealment, blinding of participants and personnel, blinding of outcome assessment, incomplete outcome data, selective outcome reporting and other sources of bias.

### Statistical Analysis

The NMA is a mixed-treatment comparison meta-analysis that simultaneously integrates both direct and indirect comparisons. For example, if a direct comparison between treatments A and C is lacking, a closed loop (namely, a network) consisting of A-B-C-(A) can be established under a Bayesian framework if treatments A and C were commonly compared with treatment B across trials, enabling the identification of the relative effect between any two treatments (22, 25). In the present work, the primary endpoint was OS in the available patient cohort, and the secondary endpoints included PFS and sAE. For outcomes for analysis, HRs and ORs along with their 95% CrI were collected or calculated for OS/PFS and sAE, respectively.

By adopting the model of the lower deviance information criterion (DIC), which indicates better feasibility (26), a Bayesian NMA of OS and PFS was performed with WinBUGS (version 1.4.3); for treatment-related sAE, it was performed with the gemtc package (version 0.8-2) (27), which was implemented in R (X64 version 3.5.1). By comparing the surface under the cumulative ranking curve (SUCRA) generated by these tools, we can obtain evidence to determine the rank probability of the competing arms to rank them as first, second, third and so on (25, 28, 29). The highest SUCRA value denotes the highest probability of ranking the best, while the lowest value denotes the highest probability of ranking worst. To fit the model, parameters were set as follows for the analyses in accordance with noninformative uniform and normal prior distributions (30): 3 chains with dispersed initial values; 100,000 iterations for each chain for a total of 300,000 in the three chains for the posterior distributions; 5000 burn-ins; and an interval of 10 iterations per chain.

Network and funnel plots were generated with STATA (version 14.0) to visually illustrate the relationships of each treatment model of the available trials and evaluate the studies for publication bias (31), respectively. The inconsistency statistic (I^2^) (32) was calculated to quantify the global inconsistency across studies (i.e., between-study heterogeneity). An I^2^ < 25% indicated low heterogeneity, whereas 25% ≤ I^2^ ≤ 50% and I^2^ > 50% were deemed to be moderate and high heterogeneity, respectively (32). Local inconsistency, which may be generated in the network, was tested by the “node-splitting” technique (33) and served as a tool in the comparison between direct and indirect evidence among the entire network (34). Statistical significance was considered at *p* < 0.05.

## Results

### Eligible Studies

**Figure 1:**
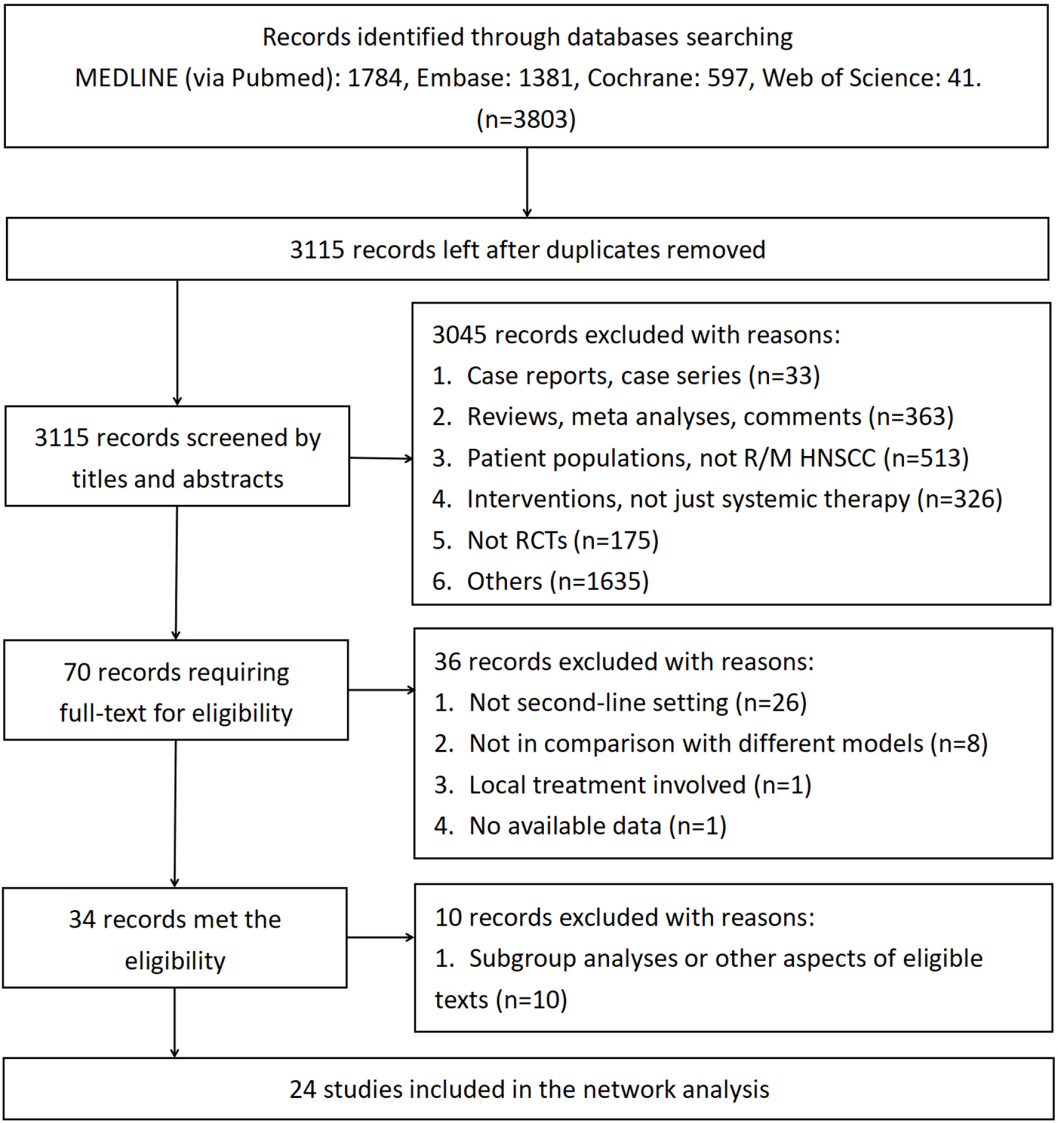
Flowchart of the literature screening. RCTs, randomized controlled trials.

**Figure 2:**
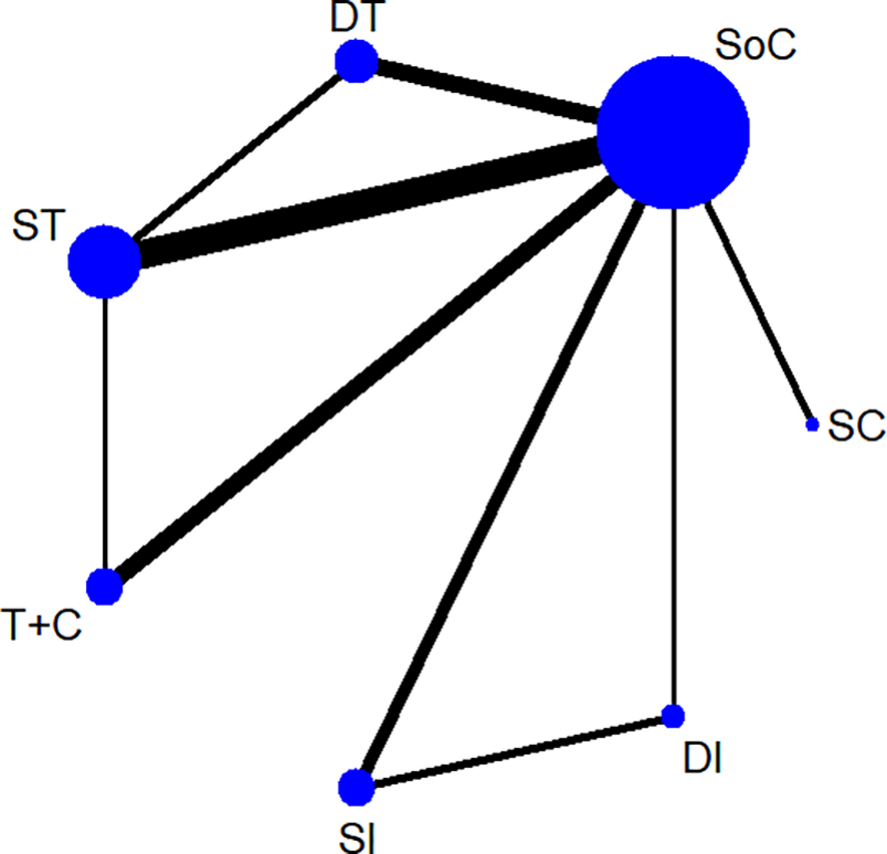
A network plot of all eligible trials. SoC, standard-of-care therapy; ST, single targeted therapy different from SoC; DT, double targeted therapy; T+C, targeted therapy combined with chemotherapy; SI, single immune checkpoint therapy; DI, double immune checkpoint therapy; SC, single chemotherapy different from SoC.

**Figure 1** demonstrates the screening of eligible studies. After searching the online databases, 3803 records were identified, with 1784, 1381, 597 and 41 recognized in the MEDLINE, Embase, Cochrane Central Register of Controlled Trials and Web of Science databases, respectively. 688 duplicate records were removed. Then, 3115 records were extensively screened *via* their titles and abstracts as extracted from the online databases. Twenty-four RCTs with 5285 participants conformed to the inclusion and exclusion criteria and thus were finally screened (9–11, 13–16, 35–51). Among the eligible studies, which consisted of treatment models of standard-of-care therapy (SoC), single targeted therapy different from SoC (ST), double targeted therapy (DT), targeted therapy combined with chemotherapy (T+C), single immune checkpoint inhibitor therapy (SI), double immune checkpoint inhibitor therapy (DI) and single chemotherapy different from SoC (SC), SoC was the most common controlled arm with the largest number participants, followed by ST, DT, T+C, SI, DI and SC, as indicated in the network plot in **Figure 2**. Of note, none of the eligible RCTs applied immunosuppressant therapy in the first-line setting and the treatment model of T+C in the second-line setting represents single targeted therapy combined with single chemotherapy. **Supplementary Table 2** summarizes the characteristics of all eligible RCTs; the HRs of the OS and PFS data and the overall sAE profiles could not be extracted from 1, 2, and 6 of these trials, respectively. As a result, 5184, 4532 and 4026 participants were finally selected for OS, PFS and sAE analyses, respectively. The network plots of the survival and sAE analyses are separately shown in **Supplementary Figure 1**.

### Quality of Evidence

**Figure 3:**
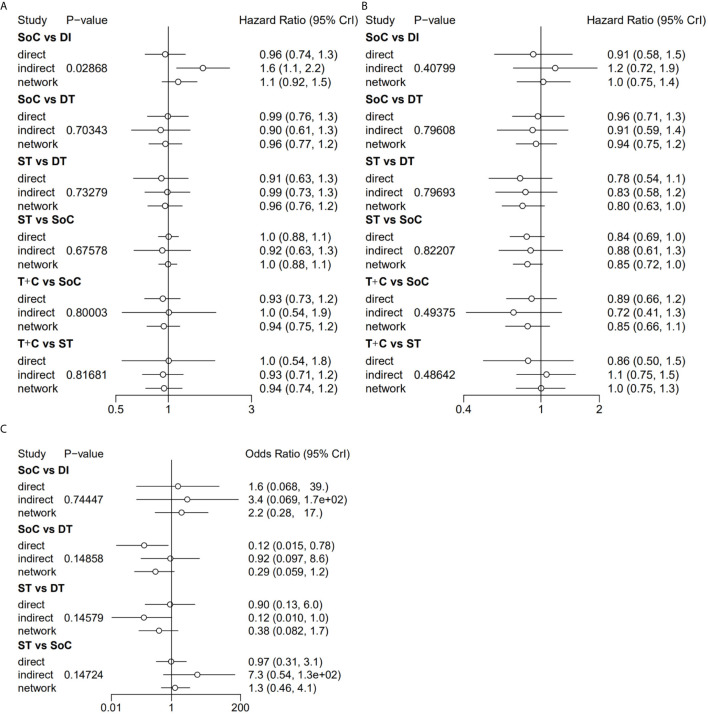
Local inconsistency of OS **(A)**, PFS **(B)** and sAE **(C)** by “node-splitting” analyses. OS, overall survival; PFS, progression-free survival; sAE, severe acute events; SoC, standard-of-care therapy; ST, single targeted therapy different from SoC; DT, double targeted therapy; T+C, targeted therapy combined with chemotherapy; SI, single immune checkpoint therapy; DI, double immune checkpoint therapy; SC, single chemotherapy different from SoC.

**Figure 4:**
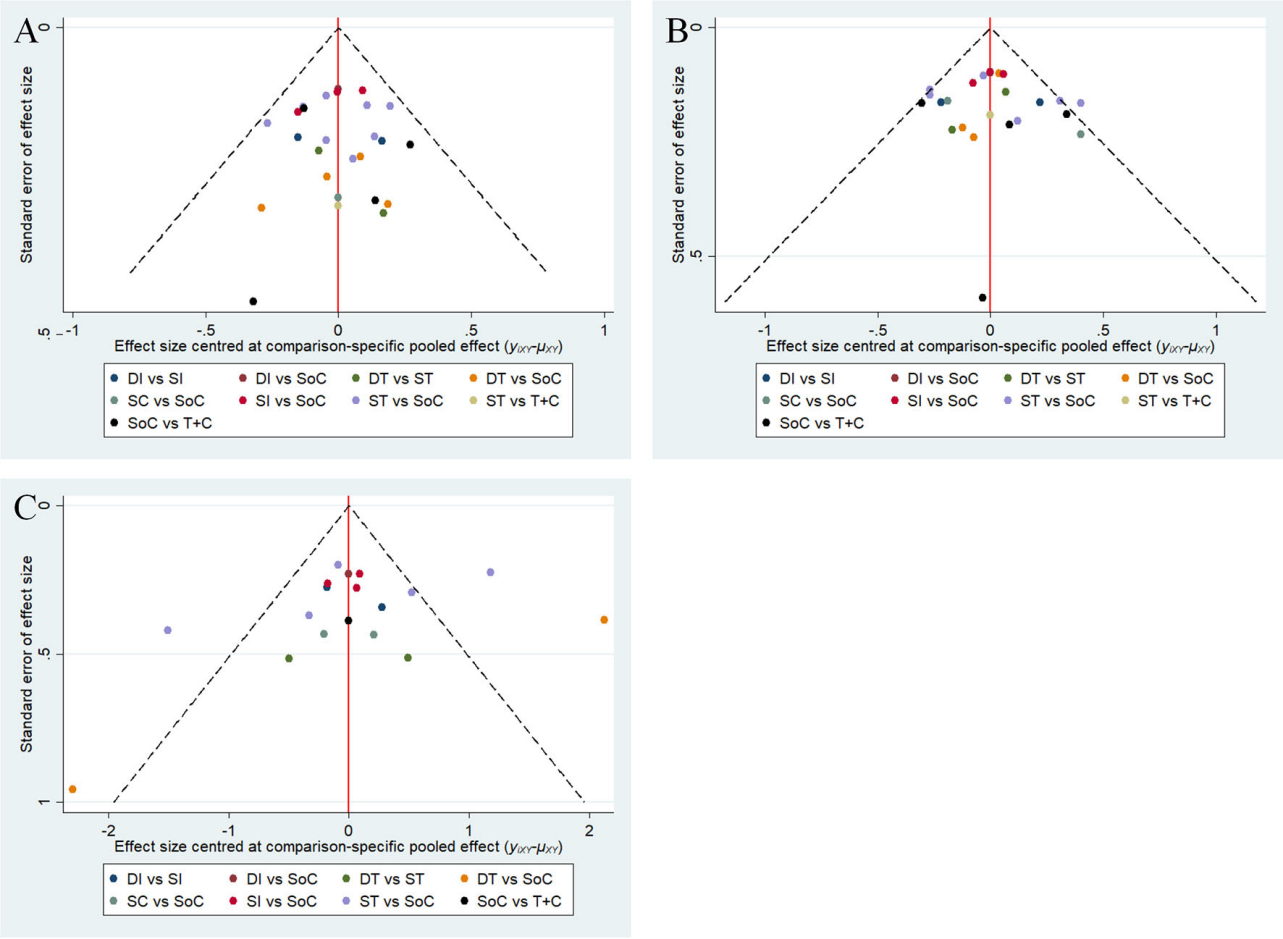
Funnel plots of OS **(A)**, PFS **(B)** and sAE **(C)**. OS, overall survival; PFS, progression-free survival; sAE, severe acute events; SoC, standard-of-care therapy; ST, single targeted therapy different from SoC; DT, double targeted therapy; T+C, targeted therapy combined with chemotherapy; SI, single immune checkpoint therapy; DI, double immune checkpoint therapy; SC, single chemotherapy different from SoC.

The assessments of all eligible studies for risk of bias are presented in **Supplementary Table 4** and **Supplementary Figure 2**; two trials were detected as having a high risk of bias. In the inconsistency analyses, all global analyses concerning OS, PFS and sAE showed low heterogeneity (I² = 14%, I² = 8%, I² = 5%, respectively). Further exploration of local inconsistency revealed that except for the *p* value between SoC and DI in the OS analysis, all other *p* values between direct and indirect evidence were greater than 0.05, suggesting that satisfactory consistency was present among the trials, as exhibited in detail in **Figure 3**. The relevant funnel plots are shown in **Figure 4**, which demonstrated the satisfactory symmetry for publication bias in the pairwise comparisons. In terms of the transitivity analysis of the present NMA, only RCTs complying with the selection criterion were strictly included, ensuring the balance for cross-study transitivity.

### NMA for Efficacy and Safety

Considering treatment efficacy, 5184 and 4532 individuals involved in 23 and 22 studies were included in the analyses of OS and PFS, respectively; their network plots are displayed in **Supplementary Figure 1**. For OS, as shown in **Table 1**, significant superiority was observed for SI when compared with ST (HR 0.88, 95% CrI 0.66 to 0.99), SoC (HR 0.76, 95% CrI 0.64 to 0.89), DT (HR 0.75, 95% CrI 0.57 to 0.96) and SC (HR 0.52, 95% CrI 0.29 to 0.91). Except for SI and SC, all treatments (DI, ST, T+C, SoC, DT) tended to be consistent, with HRs close to 1 between groups. For PFS, as described in **Table 1**, ST tended to be superior to the other treatments, although only one pairwise comparison showed a significant difference (SoC *vs* ST: HR 1.20, 95% CrI 1.08 to 1.33).

**Table 1:** Estimate results according to the network meta-analysis on OS (lower left) and PFS (upper right), along with the ranking distribution by SUCRA values (in the below arrow shape).

**SI**	1.07 (0.81, 1.41)	0.90 (0.75, 1.08)	0.93 (0.73, 1.17)	1.07 (0.92, 1.25)	1.16 (0.94, 1.42)	1.05 (0.78, 1.42)
0.85 (0.64, 1.12)	**DI**	0.86 (0.46, 1.61)	0.87 (0.45, 1.65)	1.00 (0.73, 1.38)	1.07 (0.56, 2.03)	1.03 (0.51, 2.14)
**0.81** **(0.66, 0.99)**	0.96 (0.68, 1.35)	**ST**	1.00 (0.73, 1.35)	**1.20** **(1.08, 1.33)**	1.25 (0.94, 1.61)	1.20 (0.78, 1.88)
0.80 (0.63, 1.03)	0.95 (0.65, 1.38)	0.99 (0.80, 1.23)	**T+C**	1.16 (0.97, 1.39)	1.24 (0.86, 1.78)	1.20 (0.75, 1.97)
**0.76** **(0.64, 0.89)**	0.90 (0.65, 1.24)	0.94 (0.84, 1.05)	0.94 (0.78,1.13)	**SoC**	1.08 (0.94, 1.24)	0.98 (0.75, 1.27)
**0.75** **(0.57, 0.96)**	0.88 (0.60, 1.29)	0.92 (0.74, 1.13)	0.93 (0.71, 1.22)	0.98 (0.80, 1.20)	**DT**	0.96 (0.61, 1.58)
**0.52** **(0.29, 0.91)**	0.61 (0.33, 1.16)	0.64 (0.37, 1.12)	0.65 (0.36, 1.14)	0.68 (0.40, 1.18)	0.70 (0.39, 1.24)	**SC**
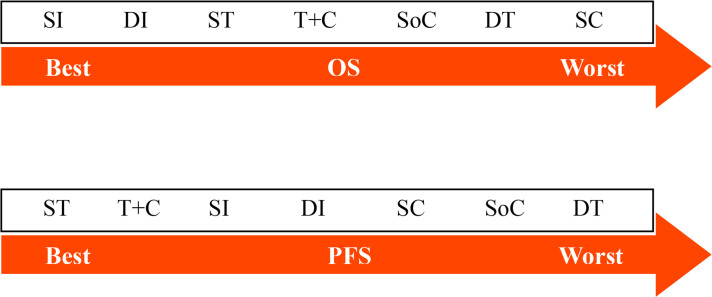						

OS, overall survival; PFS, progression-free survival; SUCRA, the surface under the cumulative ranking curve; SI, single immune checkpoint therapy; DI, double immune checkpoint therapy; SoC, standard-of-care therapy; SC, single chemotherapy different from SoC; ST, single targeted therapy different from SoC; T+C, targeted therapy combined with chemotherapy; DT, double targeted therapy. In bold: values of statistical significance. Bold values mean values of statistical significance.

In terms of safety profiles, 16 trials with 4026 participants were available for an analysis of overall sAE. The relevant network plot for sAE is shown in **Supplementary Figure 1**. **Table 2** shows that there were significantly lower incidences of sAE with SI than with SoC (OR 0.31, 95% CrI 0.11 to 0.90), ST (OR 0.23, 95% CrI 0.06 to 0.86) and DT (OR 0.11, 95% CrI 0.02 to 0.53). However, pairwise comparisons between the other treatment models demonstrated no statistically significant differences.

**Table 2:** Estimate results according to the network meta-analysis on treatment-related sAE, along with the ranking distribution by SUCRA values (in the below arrow shape).

** SI**						
0.68 (0.19, 2.42)	**DI**					
** 0.31 (0.11, 0.90)**	0.46 (0.11, 1.96)	**SoC**				
0.23 (0.04, 1.34)	0.34 (0.05, 2.57)	0.74 (0.18, 3.01)	**SC**			
** 0.23 (0.06, 0.86)**	0.34 (0.07, 1.76)	0.73 (0.33, 1.63)	0.99 (0.20, 4.99)	**ST**		
0.18 (0.02, 1.66)	0.27 (0.02, 3.02)	0.57 (0.08, 4.05)	0.78 (0.07, 8.63)	0.79 (0.10, 6.48)	**T+C**	
** 0.11 (0.02, 0.53)**	0.16 (0.02, 1.03)	0.34 (0.10, 1.12)	0.46 (0.07, 2.92)	0.47 (0.15, 1.46)	0.60 (0.06, 5.85)	**DT**
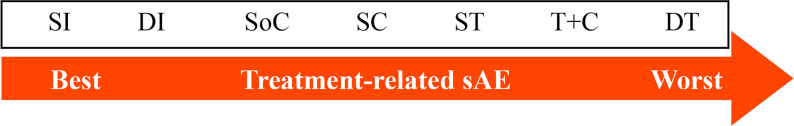						

sAE, severe acute events; SUCRA, the surface under the cumulative ranking curve; SI, single immune checkpoint therapy; DI, double immune checkpoint therapy; SoC, standard-of-care therapy; SC, single chemotherapy different from SoC; ST, single targeted therapy different from SoC; T+C, targeted therapy combined with chemotherapy; DT, double targeted therapy. In bold: values of statistical significance. Bold values mean values of statistical significance.

### Rank Probabilities

The arrows below **Tables 1** and **2** indicate the ranking distributions of safety and efficacy, respectively. Among all competing therapy models involved, as shown in **Table 1**, SI (SUCRA = 94.91%) was deemed to have the greatest probability of ranking first in terms of OS, followed by DI (SUCRA = 61.98%), ST (SUCRA = 58.08%), T+C (SUCRA = 55.69%), SoC (SUCRA = 36.34%), DT (SUCRA = 35.37%) and SC (SUCRA = 7.62%). The PFS ranking probability was not quite the same, even demonstrating outcomes opposite those of OS. The ranking sequence from best to worst for PFS were ST (SUCRA = 76.63%), T+C (SUCRA = 72.85%), SI (SUCRA = 56.37%), DI (SUCRA = 43.94%), SC (SUCRA = 37.20%), SoC (SUCRA = 36.71%) and DT (SUCRA = 26.30%). In **Table 2**, as sorted by the SUCRA values, the incidences of sAE ranked from low to high were SI (SUCRA = 89.61%), DI (SUCRA = 75.54%), SoC (SUCRA = 55.47%), SC (SUCRA = 42.41%), ST (SUCRA = 38.69%), T+C (SUCRA = 36.96%) and DT (SUCRA = 11.33%). SI performed best while DT was denoted worst.

Additionally, a cluster plot (**Supplementary Figure 3**) presenting both the efficacy and safety profiles that were defined by the SUCRA values for OS and sAE was plotted. It demonstrates the overall distribution in this setting and indicates that SI presented with the best OS and the optimal management of sAE.

### Sensitivity Analysis

For trials with small sample sizes, the relevant conclusions are not as convincing as those for larger trials. Therefore, the sensitivity analysis in this section was performed by excluding studies in which any treatment arm included no more than 50 individuals (9, 11, 35, 38, 40, 43–45, 48, 50, 51). Then, the SUCRA values for OS, PFS and sAE in the remaining trials were generated, and the rank probabilities defined by the SUCRA values were subsequently obtained. **Supplementary Table 3** denotes that SI still performed best in terms of OS and sAE, and ST was considered the optimal treatment model regarding PFS, which was consistent with the result from the initial NMA. These similar rank probabilities indicate the intrinsic robustness of the NMA to some extent, thus confirming the final results.

## Discussion

This study is the first seeking to determine the optimal second-line systemic treatment model for R/M HNSCC using an NMA and revealed that SI had been shown to be the best second-line systemic treatment model in terms of OS and sAE compared with SoC, ST, DT, T+C, DI and SC based on literature retrieval up to June 2021, without any of the eligible RCTs applying immunosuppressant therapy in the first-line setting. Furthermore, the targeted agent-involved therapeutic modalities, including ST, DT and T+C, more frequently suffered from sAE, despite the superiority of ST and T+C with regard to PFS. These findings, to some extent, could substantially guide clinical options toward favorable regimens regarding both efficacy and toxicity in the second-line treatment of R/M HNSCC patients who are unable or do not have access to immunosuppressants in the first-line setting and pave the way for future research.

For patients who have progressive disease after first-line treatment, the prognosis is poor. SoC has long been established as a cornerstone treatment (35–37, 39, 41) and was used as a control arm in many more clinical trials than other treatments, including targeted therapy (9, 10), immunotherapy (13, 14, 16), and a combination of targeted therapy and chemotherapy (45, 49). This NMA revealed that SI was the best choice for OS, while ST was best for PFS. It was not until the emergence of the CheckMate 141 and KEYNOTE 040 trials (13, 14), which compared the administration of SI (nivolumab, pembrolizumab, respectively) with SoC as a second-line treatment for R/M HNSCC, that the OS could be significantly prolonged (7.5 months *vs* 5.1 months and 8.4 months *vs* 6.9 months, respectively; both *p* < 0.05). The present NMA further confirmed the superiority of SI on OS as a second-line treatment modality.

However, the EAGLE trial, which explored the application of durvalumab and tremelimumab, proved no significant OS advantage over SoC (durvalumab *vs* SoC: HR 0.88, 95% CrI 0.72 to 1.08, *p* = 0.20; durvalumab plus tremelimumab *vs* SoC: HR 1.04, 95% CrI 0.85 to 1.26, *p* = 0.76) (16). The underlying reasons why SoC provided a longer-than-expected OS may be attributed to the differences in the choice of SoC regimen, the subsequent regimen and the imbalanced baseline clinical characteristics between groups, confounding the finding that immunotherapy resulted in non-superior survival over SoC. Nevertheless, an OS benefit was indeed observed in the durvalumab arm, with a median OS of 9.8 months in the group with programmed cell death ligand 1 (PD-L1) expression in tumor cells (TCs) > 25% versus 7.6 months for PD-L1 expression in TCs < 25%. Similarly, for pembrolizumab in KEYNOTE 040, the patients were stratified by PD-L1 combined positive score (CPS) and tumor proportion score (TPS), and compared with SoC, a significant advantage was evident in patients with PD-L1 CPS ≥ 1% (8.7 months *vs* 7.1 months, *p* = 0.0049) and in those with PD-L1 TPS ≥ 50% (11.6 months *vs* 6.6 months, *p* = 0.0014) (14). In the application of nivolumab in the Check 141 trial (13), however, the subsequent follow-up and subgroup analyses revealed that a superior OS was continuously observed in the nivolumab group regardless of PD-L1 expression, human papilloma virus (HPV) status and prior cetuximab exposure (52, 53). Hence, the level of PD-L1 expression should be measured when certain ICIs, such as durvalumab and pembrolizumab, are prescribed in the second-line treatment of R/M HNSCC, whereas the use of nivolumab in this setting may be ignored as the present evidence indicates.

In terms of PFS, we found ST to be the best choice, while DT was inferior to the other treatment models. Of note, the targeted agent-based treatment regimens, namely, ST and T+C, were the top two choices in the prolonging of PFS, but disappointingly, PFS superiority did not convert into an OS benefit but notably increased the incidence of sAE. In regard to DT, the acute toxicities were too aggressive to be tolerated, with the incidence of sAE reaching more than 50% (42), which may account for its ranking worst in terms of PFS in the present work. Notably, SI and DI rank the top two in terms of OS while it’s not the case of PFS. The potential reason why PFS is not well correlated with OS when ICIs are introduced may be ascribed to the unique mechanisms to rehabilitate or activate self-immunity toward tumors, leading to delayed clinical effects and long-term survival benefits (54). The delayed effects of ICIs also arise owing to the phenomenon of disease progression (PD) followed by either tumor shrinkage (pseudoprogression) (55, 56), or a long post-PD survival. As such, PFS exhibited only moderate-to-poor correlation with OS for ICI trials (57, 58).

For the toxicity profiles, SI was revealed to be the safest choice in this NMA, while the targeted agent-based treatment regimens were correlated with higher incidences of sAE. Attention should be paid in regard to the quality of life of patients with R/M HNSCC, especially in the setting of second-line treatments. In clinical practice, first-line treatment tends to be intense and aggressive for patients with R/M HNSCC. In both the Extreme (59) and TPExtreme trials (60), cetuximab in combination with cytotoxic agents platinum plus 5-fluorouracil with or without docetaxel was the basis utilization, which indeed caused conspicuous side effects. In the Extreme trial (59), six cycles of platinum plus 5-fluorouracil with or without weekly cetuximab both resulted in profound ≥ grade 3 overall toxicity (82% and 76%, respectively), and the maintenance of cetuximab resulted in a certain proportion of ≥ grade 3 infusion-related adverse effects, such as septic shock (7%), hypomagnesemia (9%) and skin reactions (9%). Although TPExtreme (60) made efforts to improve patients’ treatment tolerance, the incidences of sAE were still far greater than envisaged, rendering subsequent second-line treatments somewhat troublesome. As recommended by the NCCN guidelines, the second-line choice for patients with R/M HNSCC includes mainly monotherapy, such as SI, SoC, SC and ST. However, attempts have been made to introduce combination methods such as T+C or DT (11, 38–40, 43–45, 49–51). The results demonstrated that neither T+C nor DT had an OS advantage over ST or SoC; instead, sAE were much more frequent in the combination arms. This NMA again emphasizes that SI showed overwhelming superiority in terms of tolerance over the other models and provides persuasive evidence from RCTs. The other monotherapy regimens, SC and ST, were similar in terms of sAE. The evidence presented here may guide directions for strategies when making second-line treatment decisions, among which SI monotherapy should be prioritized, while combination options, such as T+C or DT, should be considered with caution.

However, inevitably, some limitations should be acknowledged for this NMA. First, not all eligible trials simultaneously reported survival data and overall sAE, resulting in an outflow of participants in some trials. At the same time, except for SoC, indirect comparisons were made for most treatment models only, so the direct evidence among all models was insufficient, and it is difficult to form sufficient closed loops in the analyses of survival and sAE. In addition, although this NMA was strictly limited to RCTs, the randomization procedures were not thoroughly illustrated, making it difficult to accurately assess the risk of bias from the included studies. In view of the intrinsic limitations of meta analyses, this work was strictly abidied by the Preferred Items for Systematic Reviews and Meta-Analyses (PRISMA) reporting guidelines as well as the extension statement of NMA (21, 22), thus making all analyses more convincing.

In summary, SI was shown to be the best second-line systemic treatment model in terms of OS and sAE. Owing to the promising survival superiority that KEYNOTE 048 demonstrates (17), pembrolizumab was approved in the first-line setting of R/M HNSCC. Thus, the subsequence choices following first-line utilization of pembrolizumab will definitely catch public attention since preferring SI in the second-line setting will encounter new problems. There will be new clinical data challenging immunosuppressant therapy in the second-line again, which is an issue worthy of further exploration and being looked forward to in the near future.

## Conclusion

This NMA, encompassing 5285 individuals from 24 trials, revealed that compared with SoC, ST, DT, T+C, DI and SC, SI achieved the best OS as well as the least sAE. SI may thus serve as the optimal second-line systemic treatment model for R/M HNSCC patients who are unable or do not have access to ICIs in the first-line setting.

## Data Availability Statement

The original contributions presented in the study are included in the article/**Supplementary Material**. Further inquiries can be directed to the corresponding authors.

## Author Contributions

Y-WY, R-HZ and T-ZY were guarantors of the entire study. Z-JZ, Y-WY, R-HZ and T-ZY built up the study concepts and designed this study. Z-JZ, FZ and W-YY collected the relevant data all needed. W-ZQ, K-L, J-HF, J-YT, and HL rechecked data and helped submit this manuscript. Z-JZ, W-YY and FZ performed the statistical analyses and wrote this paper. Y-WY, R-HZ and T-ZY revised the manuscript. All authors contributed to the article and approved the submitted version.

## Funding

This work was supported by grants from the National Natural Science Foundation of China (Grant number: 81773354) and the Key Clinical Technology of Guangzhou (Grant number: 2019ZD17).

## Conflict of Interest

The authors declare that the research was conducted in the absence of any commercial or financial relationships that could be construed as a potential conflict of interest.

## Publisher’s Note

All claims expressed in this article are solely those of the authors and do not necessarily represent those of their affiliated organizations, or those of the publisher, the editors and the reviewers. Any product that may be evaluated in this article, or claim that may be made by its manufacturer, is not guaranteed or endorsed by the publisher.
