# Supplementary material for: The Optimal Second-Line Systemic Treatment Model for Recurrent and/or Metastatic Head and Neck Squamous Cell Carcinoma: A Bayesian Network Meta-Analysis
Source: Front Immunol. 2021 Aug 2;12:719650. doi: 10.3389/fimmu.2021.719650 (PMC8367750; doi:10.3389/fimmu.2021.719650)
Supplement: Supplementary file 1 [file DataSheet_1.docx]

**Supplementary Materials**

**Supplementary Table 1. Screening strategy.**

| Item | Domains | Search terms for MEDLINE |
| --- | --- | --- |
| #1  #2  #3  #4  #5  #6  #7  #8  #9  #10  #11  #12  #13  #14  #15  #16  #17  #18 | P | ""Head and Neck Neoplasms"[Mesh]"  "Neoplasms, Head[Title/Abstract] AND Neck[Title/Abstract]) OR (Head, Neck Neoplasms[Title/Abstract])) OR (Cancer of Head[Title/Abstract] AND Neck[Title/Abstract])) OR (Head[Title/Abstract] AND Neck Cancer[Title/Abstract])) OR (Cancer of the Head[Title/Abstract] AND Neck[Title/Abstract])) OR (Upper Aerodigestive Tract Neoplasms[Title/Abstract])) OR (UADT Neoplasms[Title/Abstract])) OR (Neoplasm, UADT[Title/Abstract])) OR (Neoplasms, UADT[Title/Abstract])) OR (UADT Neoplasm[Title/Abstract])) OR (Neoplasms, Upper Aerodigestive Tract[Title/Abstract])) OR (Head Neoplasms[Title/Abstract])) OR (Neoplasms, Head[Title/Abstract])) OR (Neoplasms, Head[Title/Abstract])) OR (Neoplasms, Neck[Title/Abstract])) OR (Cancer of Head[Title/Abstract])) OR (Head Cancer[Title/Abstract])) OR (Cancer of the Head[Title/Abstract])) OR (Cancer of Neck[Title/Abstract])) OR (Neck Cancer[Title/Abstract])) OR (Cancer of the Neck[Title/Abstract]"  "Mouth Neoplasms"[Mesh]  "Mouth Neoplasm[Title/Abstract]) OR (Neoplasm, Mouth[Title/Abstract])) OR (Neoplasms, Oral[Title/Abstract])) OR (Neoplasm, Oral[Title/Abstract])) OR (Oral Neoplasms[Title/Abstract])) OR (Neoplasms, Mouth[Title/Abstract])) OR (Cancer of Mouth[Title/Abstract])) OR (Mouth Cancers[Title/Abstract])) OR (Oral Cancer[Title/Abstract])) OR (Oral Cancer[Title/Abstract])) OR (Cancers, Oral[Title/Abstract])) OR (Oral Cancers[Title/Abstract])) OR (Cancer of the Mouth[Title/Abstract])) OR (Mouth Cancer[Title/Abstract])) OR (Cancer, Mouth[Title/Abstract])) OR (Cancers, Mouth[Title/Abstract]) "  "Oropharyngeal Neoplasms"[Mesh]  "Neoplasm, Oropharyngeal[Title/Abstract]) OR (Neoplasm, Oropharyngeal[Title/Abstract])) OR (Oropharynx Neoplasms[Title/Abstract])) OR (Neoplasm, Oropharynx[Title/Abstract])) OR (Neoplasms, Oropharynx[Title/Abstract])) OR (Oropharynx Neoplasm[Title/Abstract])) OR (Oropharynx Neoplasm[Title/Abstract])) OR (Cancer of Oropharnyx[Title/Abstract])) OR (Cancer of Oropharnyx[Title/Abstract])) OR (Oropharnyx Cancers[Title/Abstract])) OR (Oropharyngeal Cancer[Title/Abstract])) OR (Cancer, Oropharyngeal[Title/Abstract])) OR (Cancers, Oropharyngeal[Title/Abstract])) OR (Oropharyngeal Cancers[Title/Abstract])) OR (Oropharynx Cancer[Title/Abstract])) OR (Cancer, Oropharynx[Title/Abstract])) OR (Cancers, Oropharynx[Title/Abstract])) OR (Oropharynx Cancers[Title/Abstract])) OR (Cancer of the Oropharynx[Title/Abstract])"  "Laryngeal Neoplasms"[Mesh]) OR (((((((((((((((((Neoplasms, Laryngeal[Title/Abstract]) OR (Laryngeal Neoplasm[Title/Abstract])) OR (Neoplasm, Laryngeal[Title/Abstract])) OR (Larynx Neoplasms[Title/Abstract])) OR (Larynx Neoplasm[Title/Abstract])) OR (Neoplasm, Larynx[Title/Abstract])) OR (Neoplasms, Larynx[Title/Abstract])) OR (Cancer of Larynx[Title/Abstract])) OR (Larynx Cancers[Title/Abstract])) OR (Laryngeal Cancer[Title/Abstract])) OR (Cancer, Laryngeal[Title/Abstract])) OR (Cancers, Laryngeal[Title/Abstract])) OR (Cancers, Laryngeal[Title/Abstract])) OR (Larynx Cancer[Title/Abstract])) OR (Cancer, Larynx[Title/Abstract])) OR (Cancers, Larynx[Title/Abstract])) OR (Cancer of the Larynx[Title/Abstract]"  "hypopharynx neoplasms[Title/Abstract] "  "Squamous Cell Carcinoma of Head and Neck"[Mesh]  ("Squamous Cell Carcinoma of Head and Neck"[Mesh]) OR ((((Squamous Cell Carcinoma of the Head[Title/Abstract] AND Neck[Title/Abstract]) OR (Squamous Cell Carcinoma, Head[Title/Abstract] AND Neck[Title/Abstract])) OR (Carcinoma, Squamous Cell of Head[Title/Abstract] AND Neck[Title/Abstract])) OR (Head[Title/Abstract] AND Neck Squamous Cell Carcinoma[Title/Abstract]))  #1 OR #2 OR #3 OR #4 OR #5 OR #6 OR #7 OR #8 OR #9 OR #10 OR #11  "Neoplasm Metastasis"[Mesh]  metastatic[Title/Abstract]) OR (Metastases, Neoplasm[Title/Abstract])) OR (Neoplasm Metastases[Title/Abstract])) OR (Metastasis[Title/Abstract])) OR (Metastases[Title/Abstract])) OR (Metastasis, Neoplasm[Title/Abstract]  "Neoplasm Recurrence, Local"[Mesh]  Local Neoplasm Recurrences[Title]) OR (Locoregional Neoplasm Recurrence[Title])) OR (Recurrences, Local Neoplasm[Title])) OR (Neoplasm Recurrences, Local[Title])) OR (Recurrence, Local Neoplasm[Title])) OR (Recurrence, Locoregional Neoplasm[Title])) OR (Local Neoplasm Recurrence[Title])) OR (Neoplasm Recurrence, Locoregional[Title])) OR (Locoregional Neoplasm Recurrences[Title])) OR (Neoplasm Recurrences, Locoregional[Title])) OR (Recurrences, Locoregional Neoplasm[Title])  #13 OR #14 OR #15 OR #16  #12 AND #17 |
| #19 | I | therapy[Title/Abstract]) OR (treatment[Title/Abstract])) OR (chemotherapy[Title/Abstract]) |
| #20 | **S** | Randomized Controlled Trial[ptyp] OR controlled clinical trial[ptyp] OR randomized[title/abstract] OR randomised[title/abstract] OR randomly[title/abstract] OR trial[title/abstract] OR phase[title/abstract]) |
| #21 | PI**S** | #18 AND #19 AND #20  Filters: from 2001/1/1 - 2021/6/1 |

**Supplementary Table 2. Charataristics of included studies.**

| First author, year | Sample size (n) ^†^ | Median age (years) ^†^ | Median follow-up time (months) | Institution | Phase | Test arm | Control arm | Study design on models | OS ^†^ | PFS ^†^ | sAE (Test arm) | sAE (Control arm) |
| --- | --- | --- | --- | --- | --- | --- | --- | --- | --- | --- | --- | --- |
| Pivot, (2001) | 93/46 | 57.9/62 | 7.4 | Multiple | NA | Nolatrexed | Methotrexate | ST vs SoC | 3.5/3.7 | 1.9/1.5 | NA | NA |
| Stewart, (2009) | 325/322 | NA | 6.2 | Multiple | III | Gefitinib | Methotrexate | ST vs SoC | 5.6/6.7(250mg)  6.0/6.7(500mg) | NA | 16/158(250mg)  34/166(500mg) | 56/159 |
| Machiels, (2011) | 191/95 | 57/58 | 6 | Multiple | III | Zalutumumab | Methotrexate | ST vs SoC | 6.7/5.2 | 2.48/2.1 | NA | NA |
| Gilbert, (2013) | 23/38 | 61/61.5 | NA | Multiple | II | Bortezomid plus Irinotecan | Bortezomid | T+C vs ST | 9.1/7.3 | 1.6/1.5 | NA | NA |
| Argiris, (2013) | 134/136 | 60.8/61.4 | 35 | Multiple | III | Docetaxel plus Gefitinib | Docetaxel | T+C vs SoC | 7.3/6.0 | 3.5/2.1 | NA | NA |
| Limaye, (2013) | 15/14 | 60/56 | NA | Multiple | II | Docetaxel plus Vandetanib | Docetaxel | T+C vs SoC | 24.1w/26.8w | 9w/3.21 | NA | NA |
| Seiwert, (2014) | 61/60 | 58/58 | NA | Multiple | II | Afatinib | Cetuximab | ST vs SoC | 8.95/11.78 | 3.25/3.75 | 32/61 | 11/60 |
| Ruzsa, (2014) | 53/53 | 58/57 | 19.3 | Multiple | II | Cetuximab plus EMD-1201081 | Cetuximab | DT vs SoC | NA | 1.5/1.9 | 30/54 | 27/54 |
| Jimeno, (2014) | 42/41 | 59/63 | NA | Multiple | II | Cetuximab plus PX-866 | Cetuximab | DT vs SoC | 211d/256d | 80d/80d | NA | NA |
| Gilbert, (2015) | 28/27 | 60/59 | NA | Multiple | II | Cetuximab plus Sorafenib | Cetuximab | DT vs SoC | 5.7/9.0 | 3.2/3.0 | 26/28 | 3/27 |
| Jimeno, (2015) | 42/43 | 62/60 | NA | Multiple | II | Docetaxel plus PX-866 | Docetaxel | T+C vs SoC | 263d/195d | 92d/82d | NA | NA |
| Machiels, (2015) | 322/161 | 60/59 | 6.7 | Multiple | III | Afatinib | Methotrexate | ST vs SoC | 6.8/6.0 | 2.6/1.7 | 127/320 | 57/160 |
| Fayette, (2016) | 59/62 | 62/62 | NA | Multiple | II | Duligotuzumab | Cetuximab | ST vs SoC | 7.2/8.7 | 4.2/4.0 | 36/59 | 31/61 |
| Ferris, (2016) | 240/121 | 59/61 | 5.1 | Multiple | III | Nivolumab | Methotrexate, Docetaxel, or Cetuximab | SI vs SoC | 7.5/5.1 | 2.0/2.3 | 31/236 | 39/111 |
| Machiels, (2016) | 53/48 | 58/57.5 | NA | Multiple | II | Cabazitaxel | Methotrexate | SC vs SoC | 5.0/3.6 | 1.9/1.9 | 21/52 | 13/45 |
| Joshi, (2017) | 46/46 | 47.5/42.5 | NA | Single | II | Cabazitaxel | Docetaxel | SC vs SoC | 115d/155d | 21d/61d | 17/46 | 16/46 |
| Soulière, (2017) | 79/79 | 59/58 | 18.1 | Multiple | II | Buparlisib plus Paclitaxel | Paclitaxel | T+C vs SoC | 10.4/6.5 | 4.6/3.5 | 62/76 | 56/78 |
| Cohen, (2018) | 247/248 | 60/60 | 7.3 | Multiple | III | Pembrolizumab | Methotrexate, Docetaxel, or Cetuximab | SI vs SoC | 8.4/6.9 | 2.1/2.3 | 33/246 | 85/234 |
| Ferrarot, (2018) | 44/47 | NA | NA | Multiple | II | Cixutumumab plus Cetuximab | Cixutumumab | DT vs ST | 5.5/5.3 | 2.0/1.9 | 12/44 | 8/47 |
| Siu, (2018) | 133/134 | 62/62(Durvalumab)  62/61(Tremelimumab) | 6 | Multiple | II | Durvalumab plus Tremelimumab | Durvalumab or Tremelimumab | DI vs SI | 7.6/6.0(Durvalumab)  7.6/5.5(Tremelimumab) | 2.0/1.9(Durvalumab)  1.9/2.0(Tremelimumab) | 21/133 | 8/65(Durvalumab)  11/65(Tremelimumab) |
| Guo, (2019) | 228/112 | 55.5/58 | 6.4 | Multiple | III | Afatinib | Methotrexate | ST vs SoC | 6.9/6.4 | 2.9/2.6 | 37/228 | 24/104 |
| Ferris, (2020) | 325/249 | 59/61(Durvalumab)  61/61(Durvalumab plus Tremelimumab) | 7.2 | Multiple | III | Durvalumab or Durvalumab plus Tremelimumab | Cetuximab, a Taxane, Methotrexate or a Fluoropyrimidine | DI, SI vs SoC | 7.6/8.3(Durvalumab)  6.5/8.3(Durvalumab plus Tremelimumab) | 2.1/3.7(Durvalumab)  2.0/3.7(Durvalumab plus Tremelimumab) | 24/237(Durvalumab)  40/246(Durvalumab plus Tremelimumab) | 240/58 |
| Kochanny, (2020) | 40/38 | 60.5/63.6 | NA | Multiple | II | Tivantinib plus Cetuximab | Cetuximab | DT vs SoC | 7.4/8.6 | 3.5/3.5 | NA | NA |
| Seiwert, (2020) | 40/40 | 60/61 | NA | Multiple | II | Temsirolimus plus Cetuximab | Temsirolimus | DT vs SoC | 177d/176d | 3.5/3.5 | 28/40 | 31/40 |

Abbreviations: ^†^, these parts were presented as test arm versus (vs or /) control arm; NA, not available; OS, overall survival; PFS, progression-free survival; sAE, severe acute events; SoC, standard-of-care therapy; ST, single targeted therapy different from SoC; DT, double targeted therapy; T+C, targeted therapy combined with chemotherapy; SI, single immune checkpoint therapy; DI, double immune checkpoint therapy; SC, single chemotherapy different from SoC.

**Supplementary Table 3. SUCRA values of ranking first for OS, PFS and sAE in the initial NMA and sensitivity analysis.**

| Treatments | SUCRA Valus of OS | | SUCRA Valus of PFS | | SUCRA Valus of sAE | |
| --- | --- | --- | --- | --- | --- | --- |
|  | Initial NMA | Sensitivity Analysis | Initial NMA | Sensitivity Analysis | Initial NMA | Sensitivity Analysis |
| SoC | 36.34 | 31.26 | 36.71 | 31.73 | 55.47 | 41.41 |
| ST | 58.08 | 49.48 | **76.63** | **75.32** | 38.69 | 36.91 |
| DT | 35.37 | 26.35 | 26.30 | NA | 11.33 | 34.57 |
| T+C | 55.69 | 51.08 | 72.85 | 52.11 | 36.96 | 22.83 |
| SI | **94.91** | **86.81** | 56.37 | 50.07 | **89.61** | **90.94** |
| DI | 61.98 | 55.02 | 43.94 | 40.77 | 75.54 | 73.34 |
| SC | 7.62 | NA | 37.20 | NA | 42.41 | NA |

Abbreviations: SUCRA, the surface under the cumulative ranking curve; OS, overall survival; PFS, progression-free survival; sAE, severe acute events; NMA, network meta-analysis; SoC, standard-of-care therapy; ST, single targeted therapy different from SoC; DT, double targeted therapy; T+C, targeted therapy combined with chemotherapy; SI, single immune checkpoint therapy; DI, double immune checkpoint therapy; SC, single chemotherapy different from SoC.

**Supplementary Table 4. Risk of bias assessment of included studies.**

| First author, year | Random sequence generation | Allocation  concealment | Blinding of participants and personnel | Blinding of outcome assessment | Incomplete outcome data | Selective outcome reporting | Other sources of bias |
| --- | --- | --- | --- | --- | --- | --- | --- |
| Pivot, (2001) | Unclear risk | Low risk | Low risk | Low risk | Low risk | Unclear risk | Unclear risk |
| Stewart, (2009) | Unclear risk | Low risk | Low risk | Low risk | Low risk | Unclear risk | Unclear risk |
| Machiels, (2011) | Low risk | Low risk | Low risk | Low risk | Low risk | Unclear risk | Unclear risk |
| Gilbert, (2013) | Unclear risk | Low risk | High risk | High risk | Low risk | Unclear risk | Unclear risk |
| Argiris, (2013) | Low risk | Low risk | Low risk | Low risk | Low risk | Unclear risk | Unclear risk |
| Limaye, (2013) | Unclear risk | Low risk | Unclear risk | Unclear risk | Low risk | Unclear risk | Unclear risk |
| Seiwert, (2014) | Unclear risk | Low risk | Unclear risk | Unclear risk | Low risk | Unclear risk | Unclear risk |
| Ruzsa, (2014) | Unclear risk | Low risk | Low risk | Low risk | Low risk | Unclear risk | Unclear risk |
| Jimeno, (2014) | Unclear risk | Low risk | Unclear risk | Unclear risk | Low risk | Unclear risk | Unclear risk |
| Gilbert, (2015) | Unclear risk | Low risk | Low risk | Low risk | Low risk | Unclear risk | Unclear risk |
| Jimeno, (2015) | Unclear risk | Low risk | Unclear risk | Unclear risk | Low risk | Unclear risk | Unclear risk |
| Machiels, (2015) | Unclear risk | Low risk | Low risk | Low risk | Low risk | Unclear risk | Unclear risk |
| Fayette, (2016) | Unclear risk | Low risk | Unclear risk | Unclear risk | Low risk | Unclear risk | Unclear risk |
| Ferris, (2016) | Low risk | Low risk | Unclear risk | Unclear risk | Low risk | Unclear risk | Unclear risk |
| Machiels, (2016) | Low risk | Low risk | Low risk | Low risk | Low risk | Unclear risk | Unclear risk |
| Joshi, (2017) | Unclear risk | Low risk | High risk | High risk | Low risk | Unclear risk | Unclear risk |
| Soulière, (2017) | Low risk | Low risk | Low risk | Low risk | Low risk | Unclear risk | Unclear risk |
| Cohen, (2018) | Low risk | Low risk | Low risk | Low risk | Low risk | Unclear risk | Unclear risk |
| Ferrarot, (2018) | Unclear risk | Low risk | Unclear risk | Unclear risk | Low risk | Unclear risk | Unclear risk |
| Siu, (2018) | Low risk | Low risk | Low risk | Low risk | Low risk | Unclear risk | Unclear risk |
| Guo, (2019) | Low risk | Low risk | Low risk | Low risk | Low risk | Unclear risk | Unclear risk |
| Ferris, (2020) | Low risk | Low risk | Low risk | Low risk | Low risk | Unclear risk | Unclear risk |
| Kochanny, (2020) | Low risk | Low risk | Unclear risk | Unclear risk | Low risk | Unclear risk | Unclear risk |
| Seiwert, (2020) | Low risk | Low risk | Unclear risk | Unclear risk | Low risk | Unclear risk | Unclear risk |

**
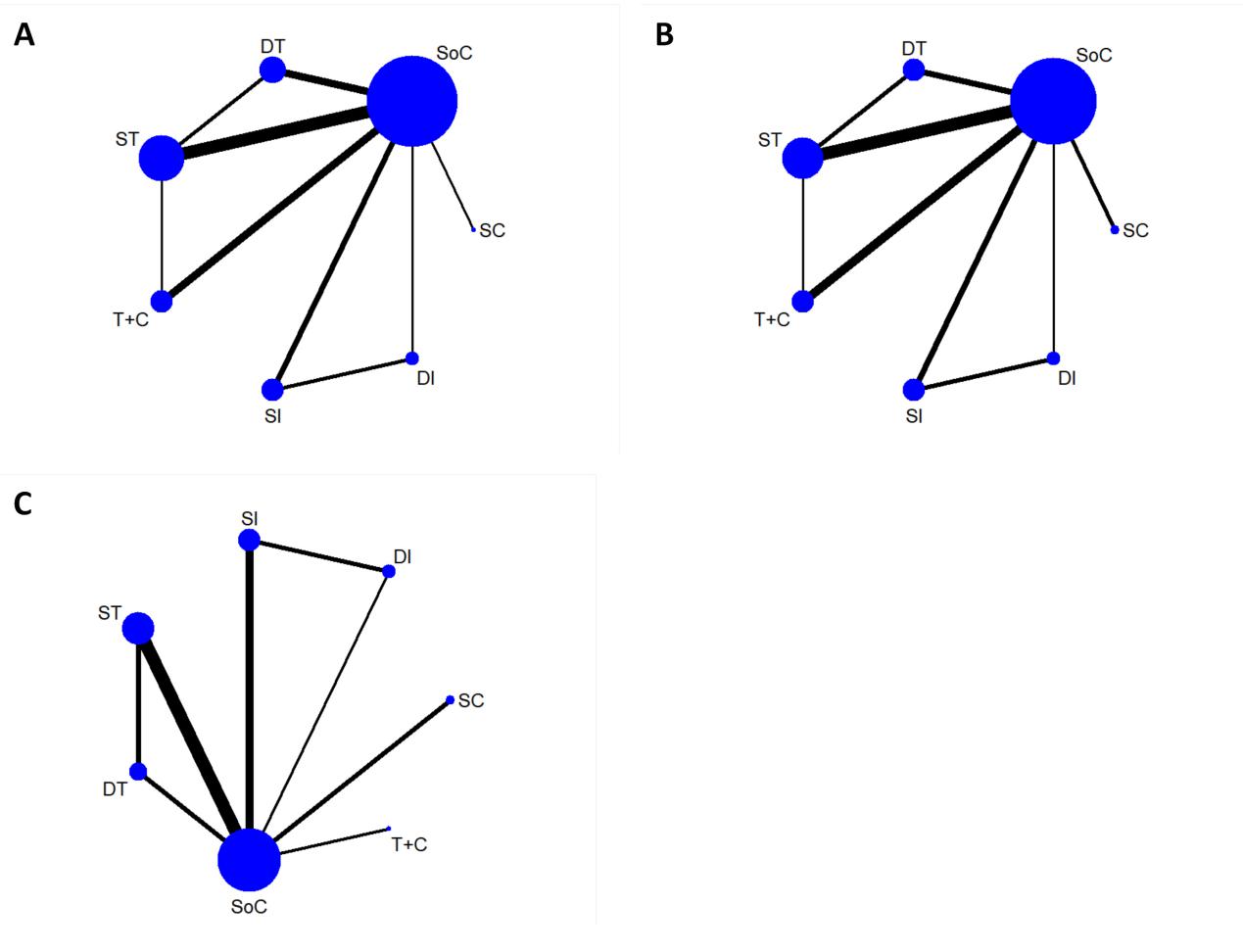
**

**Supplementary Figure 1. Network plots of OS (A), PFS (B) and sAE (C).**

Abbreviations: OS, overall survival; PFS, progression-free survival; sAE, severe acute events; SoC, standard-of-care therapy; ST, single targeted therapy different from SoC; DT, double targeted therapy; T+C, targeted therapy combined with chemotherapy; SI, single immune checkpoint therapy; DI, double immune checkpoint therapy; SC, single chemotherapy different from SoC.

**
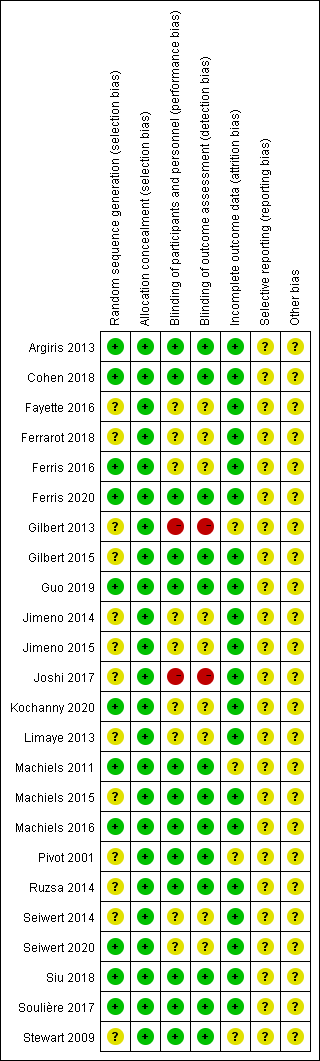
**

**Supplementary Figure 2. Summary of results from assessment of studies using the Cochrane risk of bias tool.**

**
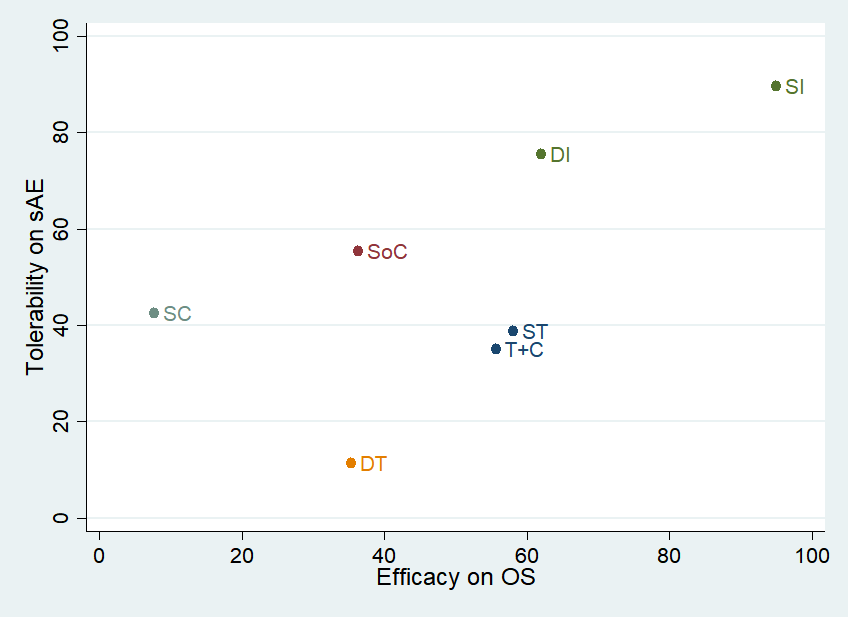
**

**Supplementary Figure 3. A cluster plot presenting SUCRA values of both OS and sAE.**

Abbreviations: SUCRA, the surface under the cumulative ranking curve; OS, overall survival; sAE, severe acute events; SoC, standard-of-care therapy; ST, single targeted therapy different from SoC; DT, double targeted therapy; T+C, targeted therapy combined with chemotherapy; SI, single immune checkpoint therapy; DI, double immune checkpoint therapy; SC, single chemotherapy different from SoC.
